# Subependymal Giant Cell Astrocytoma: The Molecular Landscape and Treatment Advances

**DOI:** 10.3390/cancers16193406

**Published:** 2024-10-07

**Authors:** Emanuela Pucko, Dorota Sulejczak, Robert P. Ostrowski

**Affiliations:** 1 Department of Neurooncology, Mossakowski Medical Research Institute, Polish Academy of Sciences, Pawinskiego 5 St., 02-106 Warsaw, Poland; epucko@imdik.pan.pl; 2 Department of Experimental Pharmacology, Mossakowski Medical Research Institute, Polish Academy of Sciences, Pawinskiego 5 St., 02-106 Warsaw, Poland

**Keywords:** subependymal giant cell astrocytoma, tuberous sclerosis complex, rapamycin, mTOR, PI3K, microRNA

## Abstract

**Simple Summary:**

Subependymal giant cell astrocytoma is a slow-growing brain tumor affecting children and young adults. Despite the characteristic molecular and clinical features, the severity of the disease varies from patient to patient, and, in addition, tumors in their early stages can be asymptomatic for a long time. Morbidity and mortality are due to the location of the tumor, which can obstruct the flow of cerebrospinal fluid, leading to progressive hydrocephalus and even death. The etiology and the response to treatment are still poorly understood due to the diversity of the background and the course of the disease. Therefore, this review aims to present and discuss the latest research on SEGA, its diagnosis, and treatment.

**Abstract:**

Subependymal giant cell astrocytoma (SEGA) is most often found in patients with TSC (Tuberous Sclerosis Complex). Although it has been classified as a benign tumor, it may create a serious medical problem leading to grave consequences, including young patient demise. Surgery and chemotherapy belong to the gold standard of treatment. A broader pharmacological approach involves the ever-growing number of rapalogs and ATP-competitive inhibitors, as well as compounds targeting other kinases, such as dual PI3K/mTOR inhibitors and CK2 kinase inhibitors. Novel approaches may utilize noncoding RNA-based therapeutics and are extensively investigated to this end. The purpose of our review was to characterize SEGA and discuss the latest trends in the diagnosis and therapy of this disease.

## 1. Introduction to Subependymal Giant Cell Astrocytoma Etiology, Clinical Presentation, and Diagnostics

Subependymal giant cell astrocytoma (SEGA) is a rare benign tumor of astrocytic origin characterized by poor growth [1]. This tumor is included among first-degree brain tumors, according to the World Health Organization (WHO) [2] and accounts for about 2% of all pediatric tumors [3]. SEGA is often diagnosed in the first or second decade of life, although it can be detected in newborns and fetuses [4]. SEGAs originate from subependymal nodules (SENs), which most often do not cause symptoms in the patient [5]. SEGAs often grow on the side of the wall of the lateral ventricle, in the area of Monro’s septum, and they can be found in the third ventricle as well [6]. Although they are benign tumors, their location and growth potential can pose a risk. Despite their small size and slow growth, when they enlarge, they may block the flow of cerebrospinal fluid, which leads to a number of different clinical symptoms, including increased intracranial pressure associated with headaches, photophobia, double vision, and ataxia [7]. Blockage of CSF passage may also lead to hydrocephalus, an increase in intracranial pressure, and possibly fatal outcomes [6]. Additionally, SEGA may also produce seizures, blurred vision, and behavioral dysfunction. Despite being grade 1 of histological malignancy, clinically aggressive cases have also been reported [6,8,9].

SEGA diagnosis involves clinical assessment and imaging techniques. Magnetic resonance imaging shows tumors as areas of contrast enhancement in the characteristic locations described above. MRI allows detection and localiza tion of the tumor(s), assessment of their size and rate of growth, and assessment of their potential to cause hydrocephalus. A variety of methods of SEGA tumor size assessment in MRI studies have been so far applied, for example, ITK-Snap (pixel clustering, geodesic active contours, region competition methods), 3D Slicer (level-set thresholding), and NIRFast (k-means clustering, Markov random fields) [10]. SEGA is a well-defined/circumscribed tumor mass on a radiology scan, be it an MRI or CT. Neuroimaging after surgery may also visualize residual tumors in cases of incomplete resection. Gadolinium enhancement and tumor growth on consecutive neuroimaging scans allow us to distinguish SEGA from SEN. MRI surveillance currently allows the diagnosis of the majority of SEGA cases even before hydrocephalus develops. It is very important that patients with SEGA diagnosed in childhood undergo scrupulous brain neuroimaging assessment on a regular basis [11,12,13].

As mentioned, SEGAs can initially be asymptomatic. However, as the tumor(s) grow, neurological symptoms may appear. Therefore, neurological examinations are very important in diagnosing SEGA and, subsequently, in patient care. Symptoms such as nausea, headaches, or visual disturbances may indicate tumor growth. Genetic testing is not routinely performed for the diagnosis of SEGA, but the detection of mutations characteristic of TSC can help in diagnosing the disease [5].

In this review, we summarize SEGA etiology and classification alongside the clinical presentation and diagnostic information in the introductory chapter. Then, we cover the neuropathological characteristics of SEGA and SEN and move to the condition principally associated with SEGA, i.e., Tuberous Sclerosis Complex (TSC). We describe TSC etiology and genetic background as well as the genetic and molecular mechanisms of SEGA formation upon TSC. The therapeutic approach chapter deals with both conservative and candidate therapies, the latter emerging from experimental and clinical studies. The pathogenetic background of SEGA and tumor biology, as well as conservative and novel therapeutic approaches, are supported by Tables and Figures summarizing the relevant information. We conclude on the insidious nature of SEGA tumors that, despite being non-malignant tumors, require novel therapies, especially for pediatric cases with unsatisfactory outcomes of standard therapy.

## 2. Neuropathological Characteristics of SEGA

SEGA is characterized by neuronal–glial structure and a low proliferation index. SEGA-forming cells are large and polygonal or epithelioid cellular in shape with abundant cytoplasm and vascular–parenchymal stroma alongside the presence of vascular calcifications [1]. As SEGA cells are capable of differentiating bidirectionally, the expression of glial and neuronal markers may coexist within the same tumor cells. Bidirectional cell differentiation may be the result of a defect in progenitor cell differentiation during brain development [14]. Thus, in descriptive terms, SEGAs are composed of ganglion-like astrocytes. These are large cells of the gemistocytic type, with abundant eosinophilic cytoplasm and a peripheral location of the nucleus and distinct nucleolus [15,16].

Part of the SEGA tumor-forming cells shows astroglial differentiation, reflected by immunoreactivity of glial fibrillary acidic protein (GFAP) and S100, whereas beta-tubulin class III protein and features of neuronal differentiation in the form of expression of neurofilament proteins and neuron-specific enolase are present in a distinct subpopulation of tumor cells [1]. Interestingly, some of the cells do not express GFAP, and some cells only weakly express neurofilaments [17]. SEGA also showed the expression of markers present in progenitor cells emerging from the periventricular zone around the lateral ventricle. Bidirectional differentiation of cancer cells may, therefore, result from a defect in the differentiation of progenitor cells during brain development [14,18]. The prototype SEGA cells are radial glial cells that play a role during embryonic development, especially upon neuronal migration towards gray matter structures in the course of maturation of the central nervous system (CNS) [19].

SEGA tumor shows variable immunoreactivity. Therefore, specific antibodies are needed to immunolabel both glial and neuronal markers of SEGA cells. TTF-1, a 38 kDa DNA-binding protein, is encoded by the homeobox gene NKX2-1 located on chromosome 14q13 and is temporarily expressed during embryonic development of the ventral forebrain [20]. Detailed animal studies have demonstrated the involvement of thyroid transcription factor (TTF-1) in cells of the midbrain and diencephalic origin of the developing brain [21,22]. TTF-1 has also been shown to be transiently expressed in some brain areas in the postnatal period [23]. It seems important that TTF-1 protein expression can be examined when diagnosing primary brain tumors of uncertain nature [24]. TTF-1 expression has been detected in brain tumors, including the pituitary gland and spindle cell oncocytoma [25]. Interestingly, TTF-1 as a marker can influence SEGA histiogenesis. Studies have revealed a sizable mRNA content of TTF-1 in ependymal and subependymal cells of the third ventricle, pointing towards the diagnostic utility of this marker in distinguishing SEGAs from its mimics, especially considering that TTF-1 immunopositivity was seen in all cases of SEGA [20].

SEGA is difficult to clearly define histopathologically because SENs and SEGA are oftentimes barely distinguishable. SENs are hyperdense, often calcified nodular masses present in the majority of TSC patients in whom they arise embryonically but also during the neonatal period. SENs are composed of spindle GFAP-positive cells intermingled with groups of balloon cells, which are often PAS and vimentin-positive. SENs originate from the subependymal zone of the periventricular region and can also be found near the caudate nucleus or the foramen of Monro. SENs are classified as a major feature for the diagnosis of TSC [26]. In addition to that, both SEN and SEGA show increased levels of cytoplasmic proteins phospho-S6K, phospho-S6, and phospho-Stat3 downstream of mTORC1 [27].

However, the size of SEN is less than 5 mm (while SEGA > 5 mm), and the growth is lacking in most instances. They are also characterized by the lack of contrast enhancement on CT and MRI, in contrast to SEGA [28,29]. SEGA, but not SEN, may manifest itself with hydrocephalus, focal neurological deficit, or symptoms of increased intracranial pressure. However, approximately 5–15% of TSC SENs transform into SEGAs [30]. Interestingly, in the analysis of the expression of Akt, Erk, and mTOR (mammalian target of rapamycin) pathways in SEN and SEGA by the team of Siedlecka and colleagues, it was shown that there is an upregulation of p-Erk, p-Mek, or p-RSK1 in SEGA but not in the SEN sample, while p-Akt, p-GSK3β, and p-PDK1 were upregulated in both SEN and SEGA from the same tuberous sclerosis disease patient [31]. Those authors proposed a research hypothesis that activation of PI3K/Akt leads to upregulation of mTOR. According to their hypothesis, it is the activation of Erk that triggers the transformation of SEN into SEGA [31]. Many authors also report that dysregulation of the mTOR pathway leads to uncontrolled cell division and tumor development [5,32,33].

## 3. Tuberous Sclerosis Complex

SEGA tumors are most often diagnosed in patients with Tuberous Sclerosis Complex, which is also known as Bourneville-Pringle disease [3]. It is a genetic disorder that eventually leads to benign tumors in various organs of the body, including the heart, kidneys, lungs, skin, brain, and heart [5]. Data show that SEGAs occur in approximately 5–20% of patients with TSC [34,35,36,37]. However, there are also cases of SEGA in patients without clinical symptoms of TSC [38,39,40], and even though it is a rare phenomenon, it is estimated that there are approximately 48 cases of patients described in the literature, counting from the time of the first patient described by Hyalmagi et al. in 1979 [41,42]. The majority of TSC cases are associated with a sporadic mutation (de novo mutation) [43,44], while a smaller number of patients inherit the mutated gene from one of their parents in an autosomal dominant manner [45]. Sporadic TSC cases more often result from TSC2 than TSC1 mutations. Those cases harbor large deletions, missense mutations, and splice-junction mutations of TSC2 (mostly exons 16, 33, and 40) and mostly small deletions and nonsense mutations for TSC1 cases (predominately exons 15 and 17) [46,47]. Familial TSC cases with biallelic inactivation of TSC1 or TSC2 are caused by their mutations (including nonsense mutations, deletions, and splice-site mutations), with more frequent than in sporadic cases involvement of TSC1 [48,49,50,51].

TSC disease is extensive, multi-organ, and very heterogeneous in terms of phenotype, manifesting itself in a wide spectrum of symptoms, including skin lesions, hyperplastic lesions within the CNS, and changes in internal organs, including the kidneys, liver, heart, and lungs [52,53]. The diagnosis of TSC is based on the presence of a pathogenic mutation or the presence of two major or one major and two minor symptoms, according to the diagnostic criteria developed by the 2021 International Tuberous Sclerosis Complex Consensus Group [54]. It is important to stress that genetic testing is recommended for all patients who cannot be diagnosed on the basis of clinical symptoms alone [55].

TSC may manifest several principal intracranial pathological entities, including cortical tubers and SEGAs [56]. SENs are small lesions located in the subependymal space of the lateral ventricle; their characteristics, as determined by histopathological analysis, resemble that of SEGA, while differences comprise smaller size, absence of growth, and absence of contrast enhancement of SEN on MRI with gadolinium compared to SEGA. Patients’ cortical tubers comprise abnormal neurons and glial cells located in the cerebral cortex resulting from abnormal cellular differentiation and disturbed neuronal migration. Cortical tubers have been linked with autism and epilepsy associated with TCS [32]. However, non-growing cortical tubers are most often clinically benign, unlike a large and growing SEGA tumor that can lead to life-threatening symptoms [57].

The International TSC Clinical Consensus Group has recommended that independent genetic identification of pathogenic variants of the TSC1 or TSC2 gene should be sufficient to diagnose tuberous sclerosis disease regardless of clinical data. Furthermore, based on two major or one major and two minor clinical criteria, a clinical diagnosis of the disease can be established [54]. Literature data show that increasingly often, patients are diagnosed prenatally using magnetic resonance imaging (MRI) or ultrasound examination, and already at this stage, the changes in fetal heart and brain related to tuberous sclerosis disease can be observed [58].

## 4. Genetic Background of TSC

Tuberous sclerosis TSC is most commonly associated with a mutation in one of two tumor suppressor genes: the TSC1 gene (which codes for hamartin), located on chromosome 9 (9q34), or the TSC2 gene (which codes for tuberin), located on chromosome 16 (16p13.3) [11,59] (Table 1). Mutations in the TSC1 or TSC2 genes are not observed in only 20% of TSC patients. Additionally, the disease in such patients without mutations has a less severe course [60]. Interestingly, isolated cases of SEGA without mutations in the TSC1/TSC2 genes caused by epigenetic changes in tuberin or hamartin have been described [61]. TSC2 gene mutations occur four times more often than TSC1 gene mutations and occur in approximately one-third of all TSC patients, but unfortunately, they also determine a more severe clinical course. Animal studies show that the TSC2 gene mutation is a more common cause of severe phenotypic features, including neurological symptoms, including epilepsy, and patients with PKD (polycystic kidney disease) and TSC2 mutations are more often exposed to early symptoms of polycystic kidney disease [6].

As already mentioned, the TSC1 gene encodes a protein called hamartin with a molecular weight of 130 kDa, and the TSC2 gene encodes tuberin with a molecular weight of 180 kDa. Both proteins form a protein complex called TSC1/TSC2 heterodimer and limit the activity of the mTORC1 (mammalian target of rapamycin complex 1-mTORC1) signaling pathway, which controls various cell functions, such as growth, survival, and proliferation, by inhibiting the small Ras GTPase homolog enriched in the brain (Rheb). When hamartin–tuberin is inactive, increased levels of active Rheb continuously activate the mTOR pathway, specifically mTORC1, and subsequently the phosphorylation of S6K1 and 4E-BP1 proteins [62].

The mTOR kinase belongs to a class of evolutionarily conserved threonine and serine kinases. mTOR is composed of two separate multi-subunit complexes called mTOR complex 1/2 (mTORC1/2). mTOR kinase catalyzes the phosphorylation of insulin growth factor receptor (IGF-1R), AKT kinase, 4E-binding protein 1 (4E-BP1), ribosomal protein S6 kinase (S6K), and transcription factor EB (TFEB). The signaling pathways that include the mTOR kinase are responsible for cell growth, survival, proliferation, cell migration, protein translation, nucleotide synthesis, lysosome biogenesis, and many other processes [63]. Unfortunately, mutations, amplifications, or deletions in genes related to mTOR or its complexes (mTORC1 and mTORC2) change the functioning of the mTOR signaling pathway and lead to uncontrolled cell proliferation, avoidance of apoptosis, and the development of neoplasms, including brain tumors [64].

Additionally, oxygen deprivation stress and hypoxia itself induce mTORC1 inhibition through AMPK activation and REDD1 induction, which results in TSC activation. In contrast, induction of DNA damage response signaling inhibits mTORC1 through the induction of p53 target genes such as REDD1, LKB AMPKβ, PTEN, and TSC2 [65].

Another multiprotein complex of mTOR, i.e., mTORC2, consists of mTOR, GβL/mLST8, Rictor (rapamycin-insensitive companion of mTOR), Protor/PRR5 (proline-rich protein 5), DEPTOR, and mSIN1 (mammalian stress-activated protein kinase-interacting protein 1) involved in the regulation of cell proliferation, survival, and migration. mTORC2 is activated by growth factors, which in turn activate several signaling pathways associated with tuberous sclerosis receptor tyrosine kinases (RTKs), IGF-1, Wnt, TNFα, inflammatory cytokines, and the Ras signaling cascade. RTK-mediated Ras signaling was found to activate the mTORC1 pathway via MAPK/ERK and its effector p90RSK [66]. The activation of the pathway may also occur through activating mutations in mTOR and mTORC1 and overexpression/amplification of mTORC1 and mTORC2 components.

**Table 1:** The genetic alterations upon TSC and SEGA.

Condition	Mutations Reported	Reference
TSC, familial	Frameshift > splicing, nonsense TSC1 mutations	[67]
TSC, familial	Lower proportion of TSC2 mutations in familial cases of TSC than in de novo cases; Dominant in frequency were frameshifts followed by nonsense mutations	[51]
TSC, familial	Identification of TSC1 mutations appears to be twice as likely in familial cases as in sporadic cases. Mutations in TSC2 are associated with more severe diseases, including seizures and cognitive dysfunction.	[50]
TSC, familial	Germline mutations in TSC1 (9q34.3) encoding hamartin and TSC2 (16p113.3) encoding tuberin	[68]
TSC, sporadic	Small TSC2 mutations > small TSC1 mutations > large TSC2 mutations	[46]
TSC, sporadic	Nonsense, missense > indels among TSC2 mutations	[48]
TSC, sporadic	Sporadic TSC cases more often result from TSC2 than TSC1 mutations	[49]
TSC, sporadic	Pathogenic variants in TSC2 > TSC1: 23% nonsense, 22% missense, 19% splice, 18% deletions, 8% large deletions, 2% in-frame deletions	[69]
SEGA; TSC-related	TSC2 gene deletions affecting the adjacent PKD1 have the highest risk of early SEGA development.	[70]
SEGA; TSC-related	TSC1 nonsense > deletions > insertions; missense or TSC2 nonsence > deletions, splice sites > missense; insertions. Also found were somatic mutations in genes involved in transcriptional and translational regulation, cell cycle regulation, signal transduction, cell adhesion, resistance to anti-cancer drugs, energy metabolism, ubiquitin–proteasome system functioning, immune homeostasis, and cytoskeleton stabilization.	[71]
SEGA-like; solitary	NF1 splice mutations	[72]
SEGA; solitary	TSC1 or TSC2 mutation limited to the tumor	[73]
SEGA; solitary	TSC2 somatic mosaic mutation, including extra-tumor tissues	[74]
SEGA; solitary	EGFR amplification, CDKN2A/B homozygous deletion, chromosomal +7/−10 alterations, and TERT promoter mutation, typical molecular abnormalities usually found in GBM, were observed.	[9]

## 5. The Mechanisms of Sega Formation in Tuberous Sclerosis

It is worth mentioning that theories regarding the origin of SEGAs are controversial partly due to a limited amount of research, including a limited set of data on the expression of the mTOR pathway in SEGA cells. Even though it cannot be concluded that a specific pathway is responsible for the formation of tumors, it can be assumed that the mTOR pathway plays a large role in the formation of SEGA [75,76]. Despite reports that showed a mutation and loss of heterozygosity in TSC2 in SEGA, one belief implying that the formation of SEGA is caused only by a mutation of the TSC1 or TSC2 gene has been challenged as being incorrect, mainly because cases of patients with SEGA without clinical symptoms associated with tuberous sclerosis and without TSC1/TSC2 mutations have been described [39,61,77]. The analysis of TSC1/TSC2 mutations in SEGA DNA from 13 patients showed that nine patients had a TSC2 mutation, two had a TSC1 mutation, and two had no mutation [78].

In the literature, there are several other hypotheses regarding the mechanisms of SEGA tumor development. One of those is that SEGA is associated with mosaicism, which quite frequently occurs in TSC (up to 15% of cases), although it may lead to SEGA without TSC as well [79,80].

On the other hand, SEGA patients may have two independent inactivating somatic mutations in TSC1 or TSC2, and thereby, both copies of the TSC1 or TSC2 gene are lost in the cells that form the tumor, but the mutations in other patients’ cells are absent [6]. Nevertheless, patients with SEGA should be carefully examined for features of TSC by physicians with expertise in TSC and subsequently monitored for an extended period of time, especially in pediatric cases [78].

Interestingly, the research revealed many genes potentially involved in the development of SEGA, including the ANXA1, GPNMB, LTF, RND3 and NPTX1 S100A11 genes, sFRP4, which may act as the effector genes in SEGA, and their expression can be modulated by rapamycin [45,81,82].

In addition, Akt phosphorylation was increased in SEGA, and a high level of proteins indicating mTOR activation was also found, including phospho-S6K, phospho-S6, and phospho-STAT3 [83]. Moreover, SEGA expressed the immunoreactive phosphorylated isoforms of MEK1/2 and ERK1/2, which in turn might indicate incorrect MAP kinase signal transduction [82].

In SEGA and in cortical tumors of patients with TSC1, there is an increased expression of growth factors and their receptors, including VEGF and HIF1α (hypoxia-inducible factor-1 α), a transcription factor that regulates the level of VEGF, which contributes to excessive cell growth and proliferation. VEGF modulates the activity of the mTOR pathway cascade, which is activated in TSCs [84].

## 6. Therapeutic Approach for SEGA Tumors

In recent years, the therapeutic approach to the treatment of SEGA has changed. Until recently, surgery used to be the standard procedure, especially in cases of acute clinical cases, but unfortunately, complete removal of the lesion was not possible in all cases [85,86] (Table 2).

Surgeries carried a risk of postoperative complications, which include headaches, memory deficits, paresis, acute hydrocephalus, intratumoral hemorrhages, cerebral infarction, secondary sepsis, meningitis, epilepsy, cardiac arrest, and lung infection leading to respiratory failure [32,90,91]. Additionally, surgical treatment of brain tumors, especially in children, was associated with postoperative neurological deficits and higher mortality compared to other age groups [92]. Another incentive to search for methods other than tumor resection was tumor recurrence, which requires reoperation and may result in further clinical complications and even the death of the patient [32,93].

SEGA tumors respond slowly and progressively to fractionated radiotherapy, similar to other low-grade intracranial tumors. One SEGA tumor’s response to intercurrent everolimus administration suggests an additive effect of radiation and that the drug could be clinically exploitable. Everolimus leads to reduced tumor volume, which facilitates more focused radiation that reduces the radiation-induced side effects as compared with pancranial irradiation. In summary, patients without hydrocephalus may be a population in which induction treatment with everolimus followed by fractionated stereotactic radiotherapy could be an alternative to surgery [12].

Recently, however, the discovery of molecules responsible for the pathogenesis of cancer has led to the introduction of targeted pharmacotherapies to SEGA treatment aimed at inhibiting various kinases and signaling pathways, including mTOR kinase [3,36,81,83,94,95,96,97,98]. For some time, recommendations on SEGA treatment assessed the effectiveness of mTOR inhibitors, but their place in the treatment strategy was variable. In 2021, recommendations for everolimus were updated, which is now recommended for people with asymptomatic SEGA growth, patients with mild and moderate symptoms, and those who do not qualify for surgery or prefer medical treatment to surgery [54].

The discovery of mTOR kinase inhibitors dates back to the 1970s, when a natural substance with antifungal properties was isolated from the bacteria Streptomyces hygroscopicus, found in the soil of the island of Rapa Nui (Easter Island, Chile), and therefore the isolated substance was named rapamycin in honor of the island of Rapa Nui. Interestingly, rapamycin, in addition to its antifungal properties, also showed anticancer activity, which was described in 1984 by Eng and colleagues [99]. The identification of rapamycin targets sparked interest in the therapeutic potential of rapamycin in anticancer therapy [99,100]. Scientists have shown that the expression of FKBP12 and FKBP51 is the rate-limiting factor that determines the response to the drug rapamycin in cell lines and tissues. This discovery triggered the synthesis of synthetic/semi-synthetic derivatives of rapamycin that were active against mTOR [101]. Sirolimus is a biochemical, functional form of rapamycin that disrupts the molecular interaction between mTOR and Raptor by targeting mTORC1 and is approved for the treatment of TSC manifestations, including SEGA. Sirolimus was also approved as an immunosuppressant used in organ transplantation and in the treatment of lymphangioleiomyomatosis. In addition to that, sirolimus is now being tested clinically as a preventive treatment for TSC [64,89]. Nab-sirolimus (sirolimus based on albumin-bound nanoparticles) showed an antitumor effect in perivascular epithelial cell tumors [102]. Studies using Nab-sirolimus are underway in patients with a variety of tumors with genetic mutations in the mTOR pathway and TSC1 and TSC2 [103].

**Figure 1:**
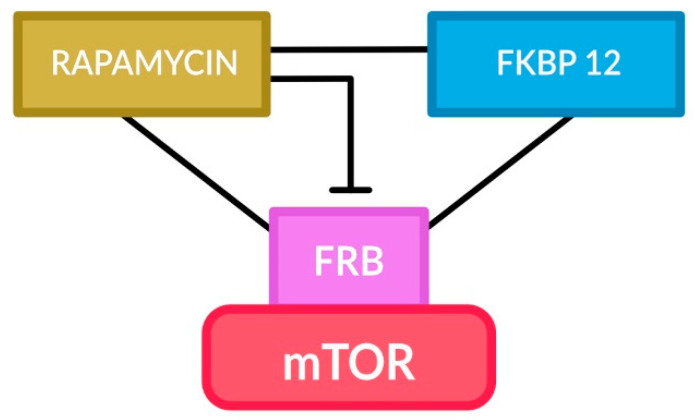
Schema depicting ternary complex formed by FRB of mTOR, FKBP12, and rapamycin leading to allosteric inhibition of mTOR (and hence its downstream targets), thereby also opposing the overstimulation of mTOR by PI3K/AKT signaling.

Everolimus was designed and synthesized as an immunosuppressive drug by replacing the H of the hydroxyl group (C-40) with a hydroxyl group [104]. Everolimus was approved for the treatment of advanced, non-functional neuroendocrine tumors of the lung or gastrointestinal tract [105]. This drug is administered orally, is metabolized by the CYP3A enzyme, and can penetrate into the brain. Everolimus treatment has been shown to reduce the size of SEGAs and the frequency of seizures [88]. Rapamycin and its derivatives, temsirolimus and everolimus, are indicated in the treatment of SEGA patients who are not eligible for surgical treatment [106]. The action of the rapamycin compound is to form a rapamycin-acceptor protein FKBP12 (FRB-FK506 binding protein 12) complex; mTOR binds to FKBP12 and rapamycin via the FKBP-rapamycin binding (FRB) domain [107,108] (see Figure 1). Thus, FKBP12 inhibits the activity of mTOR and the activity of mTOR effector proteins (p70S6K and 4E-BP1). Consequently, this causes the accumulation of cells in the G1 phase of the cell cycle and the induction of apoptosis [109]. In order to oppose the abnormal PI3K-Akt-mTOR signaling, the use of dual PI3K/mTOR inhibitors is advocated, as delineated below, among others, due to interrupting the rapalog-induced negative feedback loop that augments Akt activation [110]. Moreover, other inhibitors of mTOR kinase, i.e., ATP competitive inhibitors, are being developed to overcome the drawbacks of rapalogs, including immune suppression and feedback activation [111]. Other therapeutic attempts utilized targeting different groups of kinase, including those of the MAPK family for SEGA treatment, appear less effective. For example, inhibiting ERK signaling using the U0126 inhibitor significantly reduces SEGA cell proliferation and migration but does not affect the size of cancer cells [81,82].

Everolimus is indicated as an alternative therapy for large SEGA tumors in patients with TSC [87,112,113,114,115]. This treatment allows for an initial rapid reduction in the tumor mass and subsequent stabilization of its growth [116,117]. However, it turned out that after discontinuing the drug, the cancer process quickly recurs, prompting us to examine the safety endpoints of long-term pharmacotherapy with mTOR inhibitors [118]. Thus, continuous and long-term therapy with a risk of tumor recurrence after discontinuation of treatment can be seen as a disadvantage of the pharmacological approach to SEGA. As with any therapy, patients may experience side effects, e.g., aphthous ulcers, acne rash, inflammation of the oral mucosa, hematological complications, fever, nasopharyngitis, fatigue, otitis media, and upper respiratory tract infections [114,119]. There is also a risk of reduced insulin secretion and insulin resistance, pneumonia, sepsis, and amenorrhea, while the long-term side effects of mTOR are largely unknown [106,120,121].

Amongst studies on potential treatments for SEGA that may become standard of care treatments over time, several approaches can be named, including repositioned drugs, novel anticancer drugs, gene therapy, and novel surgical techniques (Table 3).

It is also worth mentioning that dual PI3K/mTOR inhibitors exert anticancer effects and can be considered for the treatment of SEGA brain tumors. It is known for the fact that SEGA, although assigned WHO grade 1 of histological malignancy, may resemble malignant gliomas due to its atypical histological features present at times. SEGA and malignant gliomas have a substantial portion of oncogenic signaling pathways and vulnerabilities in common, and several therapeutic agents developed for malignant gliomas originally might as well work for SEGA. This notion is supported by the results from clinical research and quite a few experimental works in cell lines derived from both high-grade and low-grade astrocytic tumors [127,133,134,135].

Paxalisib as a PI3K/mTOR inhibitor is in use for patients with newly diagnosed GBM with unmethylated MGMT promoter status following surgical resection and initial chemoradiation with temozolomide [124]. Paxalisib has entered a clinical trial for diffuse midline glioma as a monotherapy or in combination with ONC201, a TRAIL inducer [122,136]. Paxalisib can cross the blood–brain barrier and inhibit PI3K, a master regulator of neoplasm cell growth and cell division activated in SEGA; hence, it has the potential for the treatment of this astrocytoma [123].

Apitolisib (GDC-0980), a novel dual PI3K/mTOR inhibitor of mTORC1/2 and PI3K class I tested in solid tumors, including those of the brain, inhibits growth and induces apoptosis in human glioblastoma cells. Apitolisib (GDC-0980) induces time- and dose-dependent cytotoxicity and apoptosis in the tested A-172 and U-118-MG GBM glioma cell lines; the strongest apoptosis induction was demonstrated in the A-172 line after 48 h of incubation with 20 μM GDC-0980, where 46.47% of cells were apoptotic. Researchers found that dual PI3K/mTOR blockade by GDC-0980 significantly suppressed human GBM cell survival and induced apoptosis [137].

Voxtalisib has demonstrated synergistic effects with low-intensity pulsed ultrasound to inhibit tumorigenesis in glioblastoma’s CSCs while inhibiting PI3K/AKT/mTOR signaling [138]. Others have demonstrated synergistic effects of voxtalisib in combination with temozolomide and radiotherapy for glioma [139].

A promising drug that has been shown to inhibit the mTOR protein kinase is metformin, which is used in the treatment of type 2 diabetes. Interestingly, metformin has also shown anticancer properties [140]. One research group conducted a study where they determined the effect of metformin in patients with tuberous sclerosis. It was observed that patients taking metformin had a reduction in SEGA tumor volume compared to placebo and a reduction in the frequency of epileptic seizures. It is likely that the beneficial effect is mainly due to the inhibitory effect of metformin on the mTOR pathway via activation of adenosine monophosphate-activated protein kinase (AMPK) [130]. Importantly, metformin penetrates the blood–brain barrier and is distributed in many areas of the brain after oral administration [141,142]. Moreover, metformin does not interact with the cytochrome p450 system; therefore, it is unlikely to interfere with the metabolism of other mTOR inhibitors, including everolimus and rapamycin. It has been shown that the metabolism of metformin is not disturbed by antiepileptic drugs such as cannabidiol or carbamazepine [130,143,144].

**Figure 2:**
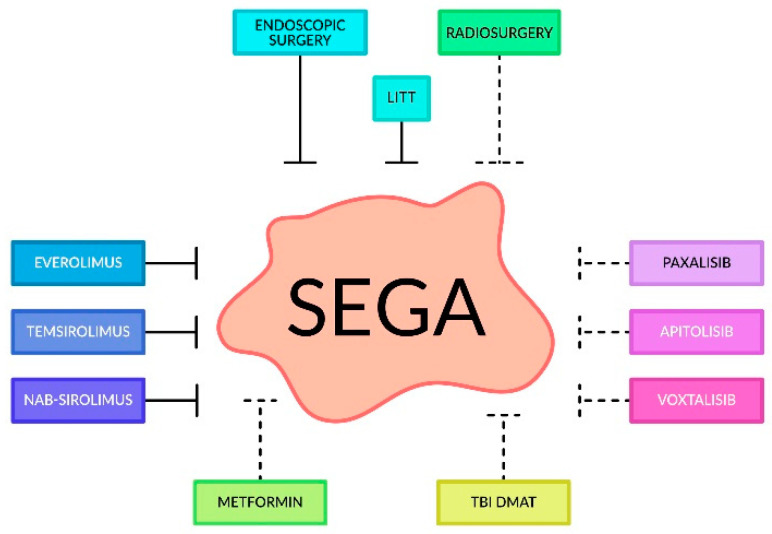
A summary of established (left and top boxes) and investigational (right and bottom boxes) therapeutics for SEGA. The latter is currently growing in number due to novel emerging therapies, including non-coding RNA-based therapeutics and nanocarrier technologies.

CK2 kinase inhibitors are also interesting candidate compounds for SEGA treatment, which have shown a reduction in cell viability and proliferation of the T98G malignant glioma cell line and the SEGA cell line, derived from a pediatric case of tuberous sclerosis complex (TSC). Cell cultures were incubated with selected CK2 inhibitors, including 4,5,6,7-tetrabromo-1H-benzimidazole (TBI), 2-dimethylamino-4,5,6,7-tetrabromo-1H-benzimidazole (DMAT), and 4,5,6,7-tetrabromo-1H-benzotriazole (TBB). Studies have shown that the tested CK2 inhibitors reduce cell growth and viability, but the strongest cytotoxic effect on SEGA and T98G cells was caused by the TBI inhibitor [127]. Promising compounds appear to be pentabromobenzyl isothiourea bromide derivatives, which cause a reduction in cell viability and proliferation of the T98G malignant glioma cell line and the SEGA cell line, derived from a pediatric case of TSC. Studies have shown that among those compounds, the best anti-proliferative effect was demonstrated by the compound ZKK13 [145]. The mainstay of current treatment options for SEGA is depicted in Figure 2.

## 7. Non-Pharmacological Treatment Modalities and Biopharmaceuticals

It is worth considering other treatment methods for SEGA, e.g., endoscopic removal of tumors. The advantage of this method is the possibility of adding a septostomy to the tumor resection, but the disadvantage is that this method is applicable to tumors less than 3 cm in size and the wide attachment of the tumor to the basal ganglia [146].

Recently, laser methods have been substantially developed, including laser interstitial thermal therapy (LITT). Amongst the limitations of its use are tumor size < 2 cm and wide attachment of the tumor to the base. Moreover, active hydrocephalus is a contraindication to LITT in SEGA due to a risk of acute hydrocephalus and swelling of the basal ganglia associated with LITT [128,129,146,147]. Thus, although LITT is a promising method, there are no long-term treatment results so far [128,148].

Another form of therapy is the use of radiosurgery using the Gamma Knife™ method, which may have the effect of reducing the size of the SEGA tumor, but unfortunately, this therapy carries an additional burden, which is the risk of developing secondary radiation-induced tumors. Moreover, such therapy is not suitable for large tumors accompanied by hydrocephalus [94,132,148].

In recent years, research advances in SEGA molecular biology have also provided the background for emerging novel therapies. Gene therapy on lentivirus restoring human TSC1 reduced growth and proliferation of TSC1-deficient neural cells. TSC1 replenishment, along with Rapamycin, further decreased proliferation and growth in TSC1-deficient tumors in mice [126].

Moreover, alterations of non-coding RNAs that may result in the dysregulation of matrix metalloproteinases (MMPs) and their endogenous tissue inhibitors (TIMPs) have been found in SEGA cells [131]. MMP/TIMP system abnormalities have been implicated in tumor recurrence and local neuroinflammation of SEGA tumors, among others [131]. Since miR-320d was found to be lowly expressed in SEGA cells, this miRNA or its mimetics could be utilized in prospective therapies. Although the disturbed MMP/TIMP system is not specific for SEGA, it can be controlled by different sets of non-coding RNAs across different tumor types, hence the importance of revealing the exact mechanism [149,150,151,152]. In addition, small non-coding RNAs have been implicated in the control of DNA replication, cell cycle, protein degradation, immune and hypoxic responses, as well as p53, PI3K/Akt, and MAPK signaling of SEGA cells, although further studies are needed to devise therapeutic systems based on such knowledge [125]. To this end, nanotechnologies may come in handy in overcoming the limitations in the clinical translation of ncRNA and other therapies as well [153,154,155,156].

In summary of the established and prospective treatments for SEGA, gross total resection of SEGA can be curative. Moreover, novel minimally invasive surgery techniques offer an option to reach a tumor such as SEGA with fewer side effects and complications and speedy recovery as compared to older techniques. However, with respect to surgery, there is a risk of a tumor growing back if not suppressed with chemotherapy with rapalogs.

Several authors, however, postulated a decreased role of surgery reserved for refractory cases because of favorable responses to mTOR inhibitors in terms of tumor suppression along with the betterment of clinical symptoms. Others imply that fractionated radiotherapy should be firmly included in the treatment of SEGA because the tumor slowly but progressively responds to fractionated radiotherapy. On the other hand, rapalog treatment cessation carries a risk of regrowth, as does the aftermath of radiotherapy. Thus, the combined therapies based on the above approaches and tailored to a particular patient appear to be optimal treatments until the beneficial effects of novel therapies are substantiated.

## 8. Conclusions

It should be emphasized that the growth of a histologically benign SEGA tumor is often associated with severe complications, including epilepsy, increased intracranial pressure, and hydrocephalus, which may lead to serious neurological deterioration and even death of the young patient. Hence, the indications for surgical interventions are solid. Preoperative administration of mTOR inhibitors to large SEGAs and tumors occurring in deep, hard-to-reach brain structures helps reduce tumor size and enable complete resection. Patients with acute obstructive hydrocephalus who have clinical symptoms of increased intracranial pressure are often treated with the combination therapy of surgery and mTOR inhibitors. They benefit from such neoadjuvant therapy based on the use of mTOR inhibitors because it reduces the size of the tumor, thus facilitating surgery and reducing the number of postoperative complications [157]. In the future, the design and synthesis of new and specific inhibitors of the mTOR pathway or mTOR-related pathways may eliminate cancer cells alone or in combination with chemotherapy drugs and immunotherapeutic agents.

It is worth emphasizing again that Nab-sirolimus (albumin-bound sirolimus nanoparticle) has been approved by the FDA for clinical use against human cancers [158]. High hopes are invested in the nanoformulation of mTOR signaling pathway inhibitors for improved effectiveness towards SEGAs and limited toxicity to healthy tissues. The new discoveries and inventions, including genes involved, therapeutic compounds, and treatment procedures, will provide grounds for significant improvement in SEGA treatment, as once the discovery of the TSC1 and TSC2 genes, rapamycin, and acting components of the mTOR pathway led to the creation of new treatment opportunities for patients with tuberous sclerosis.

## Figures and Tables

**Table 2:** Results of surgery and chemotherapies of SEGA.

Therapeutic Approach for SEGA Tumors	Effect of Therapy	References
Surgery	After surgical excision, the tumor may grow back.	[32]
Radiotherapy	SEGA responds slowly and progressively to fractionated radiotherapy.	[12]
Chemotherapy; Everolimus (mTOR kinase inhibitor)	35% of patients had at least 50% reduction in SEGA volume after 6–9 months of treatment with everolimus.	[54,87,88]
Chemotherapy; Sirolimus (mTOR kinase inhibitor)	Reductions in SEGA volume of treatment with sirolimus	[89]

**Table 3:** Investigational therapies for the treatment of SEGA.

Drug/Therapeutic Modality	Experimental/Clinical Study	Major Outcomes	References
Dual PI3K/mTOR inhibitors	clinical studies	May provide survival benefit over standard care for gliomas; to be determined for SEGA	NCT05009992, NCT03970447 [122,123,124]
ERK inhibitor	primary human derived SEGA culture	Decreased proliferation in a similar manner to treatment with rapamycin	[125]
Gene therapy	SEGA-like cell line	Recombinant lentivirus encoding human TSC1 restored the TSC1 level	[126]
Inhibitors of kinase CK2: 4,5,6,7-tetrabromo-1H-benzimidazole (TBI); 2-dimethylamino-4,5,6,7-tetrabromo-1H- benzimidazole (DMAT); 4,5, 6,7-tetrabromo-1H-benzotriazole (TBB)	cell lines established from human SEGA tumor	Reduced SEGA cell growth and viability	[127]
Laser-induced interstitial thermotherapy	clinical studies	Tumor shrinkage; less invasive surgical alternative to open resection of SEGAs	[128,129]
Metformin	clinical study	The effect of metformin on reducing SEGA volume was observed in patients	[130]
MicroRNA-320d mimic	cell culture	Ameliorated MMP/TIMP proteolytic system, dysregulated in SEGA	[131]
Radiosurgery	clinical study	Reducing the size of the SEGA tumor	[132]
